# Commentary: Attentional control and the self: The Self Attention Network (SAN)

**DOI:** 10.3389/fpsyg.2016.01701

**Published:** 2016-11-03

**Authors:** Giuseppina Porciello, Ilaria Minio-Paluello, Ilaria Bufalari

**Affiliations:** ^1^Department of Psychology, “Sapienza,” University of RomeRome, Italy; ^2^Social and Cognitive Neuroscience Laboratory, IRCCS Fondazione Santa LuciaRome, Italy; ^3^Department of Psychology of Developmental and Socialization Processes, “Sapienza,” University of RomeRome, Italy

**Keywords:** enfacement, engazement, self-gaze, multisensory integration, self-recognition, attentional reorienting

In their Discussion Paper, Humphreys and Sui (2015) review recent data on the relation between self-bias and attention and bring evidence that self-related stimuli, after a simple association, are able to alter the salience of neutral stimuli which is usually a prerogative of monetary and food reward (O'Doherty et al., 2004; Panasiti et al., 2015; Trilla Gros et al., 2015).

The authors review own-name effects, own-face effects and self-biases in associative matching and propose the “Self Attention Network” (SAN), a network model in which dorso-lateral prefrontal cortex (DLPFC) and intra-parietal sulcus (IPS) exert top-down attention-mediated control over left posterior superior temporal sulcus (LpSTS) and ventro-medial prefrontal cortex (vmPFC) which are instead respectively linked to bottom-up orienting of attention and self-related processing.

Here we would like to contribute to the SAN by suggesting that, in addition to considering the behavioral and neural mechanisms that occur when processing supra-modal self-related stimuli, the model would benefit from taking into account the plastic body-centered representation of the self (Maister and Farmer, 2015) and its effects on attention. Specifically, we would like to speculate on how SAN adapts to situations where bodily self-representation is challenged by experimental manipulations that are able to blur self-other distinction, such as shared visuo-tactile stimulation.

Our own face is one of the most important features that define the self and it has a robust representation and a special status in the human cognitive and neural systems (Keenan et al., 2003; Devue and Brédart, 2011). Self-capture and self-advantage effects while processing the self-face, have been extensively found (Tong and Nakayama, 1999; Brédart et al., 2006; Devue et al., 2009). In addition, self-face recognition is linked to the activity of a partially dedicated brain network (Devue and Brédart, 2011) and specific electrophysiological activity (Tacikowski and Nowicka, 2010).

Nevertheless, recent studies show that self-face recognition may be inherently plastic (Tsakiris, 2008; Sforza et al., 2010) as it can be modified by simple visuo-tactile Interpersonal Multisensory Stimulation (IMS) (see Porciello et al., 2016; Sel et al., 2016a for visuo-cardiac IMS). In fact, experiencing tactile stimuli on one's face while seeing synchronous tactile stimulation delivered on the face of another individual, induces changes to self-face representation: a bias in attributing the other's facial features to the self and the illusory experience of looking at oneself in the mirror (Enfacement). At the neural level such IMS modulates the activity of unimodal (inferior occipital gyrus, IOG) and multimodal (right temporo-parietal junction, TPJ and IPS) areas (Apps et al., 2015), both involved in different aspects of self-consciousness: self-location, self-identification, and first person perspective (Blanke, 2012). In particular, we suggested (Bufalari et al., 2015) that TPJ detects the mismatch between self/other tactile sensations while IPS solves the conflict between felt and observed stimuli by integrating multisensory congruent stimuli and remapping the space around the face which ultimately results in an updated self-face representation and in the illusory experience of looking at oneself in the mirror. In line with this view, recent electrophysiological evidence (Sel et al., 2016b) demonstrates that self-specific mismatch detection mechanisms exist in the brain and along with Apps et al. (2015) neuroimaging data, support the idea that self-processing follows predictive coding's principles (Friston, 2009). In such theoretical account multimodal areas update self-representation in order to minimize the surprise generated in unimodal areas by the synchronous IMS (Apps and Tsakiris, 2014). In addition to changing self-face representation, synchronous visuo-tactile IMS is also able to influence basic perceptual processes, such as detection of facial tactile stimuli (Cardini et al., 2013), and importantly higher level attentional mechanisms triggered by self-related face stimuli. Indeed, we recently measured the attentional capture exerted by the self-face and by a friend's face after participants underwent experimental synchronous and control asynchronous IMS (Porciello et al., 2014b). Participants performed a gaze-following task in which they had to look in the direction signaled by an imperative cue while ignoring distracting stimuli, i.e., either the self or a friend's face gazing toward the correct or the wrong direction. After asynchronous IMS, the distracting power of directional gaze is higher when embedded in one's own face than in a friend's face, confirming that self-similarity enhances the degree to which gaze orients attention (Hungr and Hunt, 2012). However, after synchronous IMS, namely when other's facial features are assimilated in the self-face representation (Tajadura-Jiménez et al., 2012), the distracting power of self-gaze vanishes and becomes no more distracting than a friend's face. Synchronous IMS can therefore cancel self-gaze attentional capture resulting in the Engazement effect (Porciello et al., 2014b). At the neural level, we hypothesized that Engazement may rely on the interaction between brain circuits involved in self-recognition (i.e., occipito-frontal and parietal regions, Kircher et al., 2001; Platek et al., 2008), multisensory integration and self/other distinction (i.e., TPJ and IPS, Apps et al., 2015) with those involved in reflexive shifts of attention (i.e., dorsal and ventral fronto-parietal networks, Corbetta et al., 2008; Callejas et al., 2014).

In light of the dynamic causal model developed by Humphreys and Sui (2015), we suggest that IMS-induced plasticity of self-face representation may change attentional capture related to self-gaze (i.e., make self-face stimuli less salient) via the activity of ventral (including the specific portion of TPJ connected with the PFC and the insula, Mars et al., 2012) and dorsal fronto-parietal networks which are respectively involved in bottom-up orienting (Corbetta et al., 2008; Mars et al., 2012) and in inhibitory control (Grosbras et al., 2005; Klein et al., 2009; Cazzato et al., 2012; Porciello et al., 2014a) of attention.

In particular, IMS-induced plastic change in self-face representation may reduce the activation of the two ventral SAN's nodes, namely vmPFC, which represents the self and the saliency of self-related stimuli, and posterior STS, which triggers bottom up orienting of attention toward self-related stimuli. Consequently, the dorsal fronto-parietal attentional network, including DLPFC and IPS, has to exert less control to inhibit automatic orienting of attention (e.g., gaze-following behavior) toward stimuli that, after the inclusion of other's features, are no longer coded as self-related.

To sum up, we suggest that the neurocognitive interaction between self-bias phenomena and attention passes through a basic and fundamentally plastic representation of the bodily self that may follow predictive coding rules.

## Author contributions

GP, IM-P, and IB have made substantial, direct, and intellectual contribution to the work, wrote the manuscript and approved it for publication.

### Conflict of interest statement

The authors declare that the research was conducted in the absence of any commercial or financial relationships that could be construed as a potential conflict of interest.
